# The customized prosthesis for lateral unicompartmental knee arthroplasty in the treatment of malunion of a Hoffa fracture of the distal femur: A case report

**DOI:** 10.1016/j.ijscr.2025.111231

**Published:** 2025-03-28

**Authors:** Cunxiang Ma, Qing Liu, Hangyu Gu, Teng Zhang, Junqiang Wang, Wei Han

**Affiliations:** a Department of Orthopaedic Trauma, Beijing Jishuitan Hospital, Capital Medical University, Beijing, China; b Department of Knee Preservation Surgery, Beijing Jishuitan Hospital, Capital Medical University, Beijing, China; c Seventh Clinical Medical College of Capital Medical University, Beijing, China; d Beijing Jishuitan Orthopaedic Robot Engineering Research Center Co., LTD, Beijing, China

**Keywords:** Hoffa fracture, Malunion, Unicompartmental knee arthroplasty, Customized prosthesis, Case report

## Abstract

**Introduction:**

Postoperative malunion following distal femoral Hoffa fractures is rare yet challenging. We present a novel approach using customized lateral unicompartmental knee arthroplasty (UKA) to address malunion with traumatic osteoarthritis, emphasizing functional restoration.

**Case presentation:**

A 52-year-old male presented with persistent right knee pain (VAS score: 7/10), restricted range of motion (ROM: 0°–60° flexion), and gait instability two years after open reduction and internal fixation (ORIF) of a lateral Hoffa fracture. Imaging confirmed malunion of the fracture with traumatic osteoarthritis. A customized lateral femoral condyle prosthesis was designed using three-dimensional Computed Tomography (CT) reconstruction and implanted via a lateral parapatellar approach. Postoperative imaging (8–12 weeks) revealed optimal alignment and resolved fracture gaps. At 12 weeks, pain resolved (VAS: 1/10), ROM improved to 0°–125°, and Knee Society Score reached 85/100, with no complications.

**Clinical discussion:**

Malunion after Hoffa fracture fixation is uncommon. Traditional revision ORIF may fail due to bone loss, while UKA preserves healthy compartments by restoring biomechanics. Customized implants address anatomical complexity, though long-term efficacy requires further study.

**Conclusion:**

Customized UKA offers a viable solution for Hoffa fracture malunion with traumatic osteoarthritis, prioritizing joint preservation. This approach highlights the potential of patient-specific implants in complex orthopaedic salvage.

## Highlights

• The customised prosthesis was used for unicompartmental replacement of the lateral femoral condyle.
• Lateral unicompartmental knee arthroplasty was performed for lateral condylar Hoffa fractures.
• Unicompartmental arthroplasty of the lateral femoral condyle preserves the lateral cortical bone and maintains the attachment point of the lateral collateral ligament.

## 1 Introduction

In 1869, Busch first reported a coronal plane fracture of the posterior half of the lateral condyle of the femur during a cadaveric knee dissection [1]. In 1888, Hoffa referenced Busch's illustration of this type of fracture in his book but did not credit the source [2]. Subsequently, in 1977, Letenneur et al. classified this type of fracture and named it the Hoffa fracture, specifically identifying it as a fracture of the coronal plane of the posterior half of the lateral femoral condyle [3]. Today, fractures of the coronal plane affecting the posterior half of the medial, lateral, or medial epicondyle of the femur are commonly referred to as Hoffa fractures [4,5]. These fractures are categorized as rare occurrences, constituting approximately 8.7 % to 13 % of all distal femur fractures [6].

Research indicates that lateral condylar fractures are more prevalent than their medial counterparts, accounting for roughly 78 % to 85 % of Hoffa fractures [7]. The fundamental principles of treating Hoffa fractures revolve around anatomical reduction, stable fixation, and early mobilization. Incisional reduction coupled with internal fixation is the preferred treatment for displaced Hoffa fractures and is also applicable for non-displaced cases [8]. However, the potential for re-revision presents significant challenges, often stemming from issues such as internal fixation failure and inadequate healing [9]. Therefore, managing Hoffa fractures remains a complex task, necessitating the exploration of more effective treatment options and strategies in future research and practice.

In recent years, advancements in biomaterials and computer-aided design technology have led to the increasing use of customized prostheses as innovative alternatives in various types of joint surgeries [10]. Compared to standard prostheses, customized prostheses are more adept at conforming to the unique anatomical characteristics of individual patients, thereby providing optimized support for knee joint biomechanics [11]. This adaptation may enhance functional recovery and mitigate postoperative pain [12].

According to our literature review, no study has reported the use of customized prosthesis in the treatment of Hoffa fractures. Following Surgical CAse REport (SCARE) guidelines, we presents a case of a Hoffa fracture of the lateral femoral condyle in a patient who underwent lateral UKA [13]. The procedure involved preservation of the lateral cortex and the application of a customized prosthesis, performed due to postoperative malunion and traumatic arthritis resulting from the Hoffa fracture of the distal right femur. This surgical approach offers anatomical advantages, provides immediate stability, and facilitates early mobilization and partial weight-bearing activities for the patient.

## 2 Case presentation

**Fig. 1:**
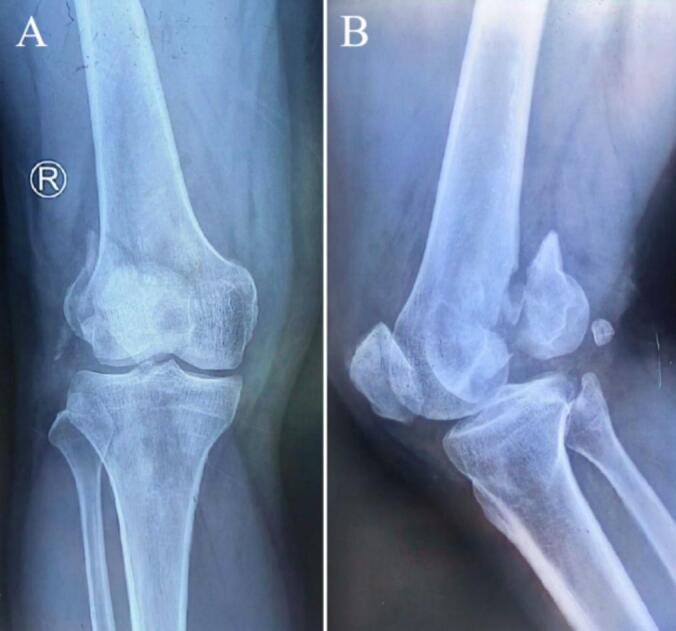
The X-ray images of the right knee after fracture. (A) Antero-posterior (AP) view of the right knee. (B) Lateral view of the right knee.

**Fig. 2:**
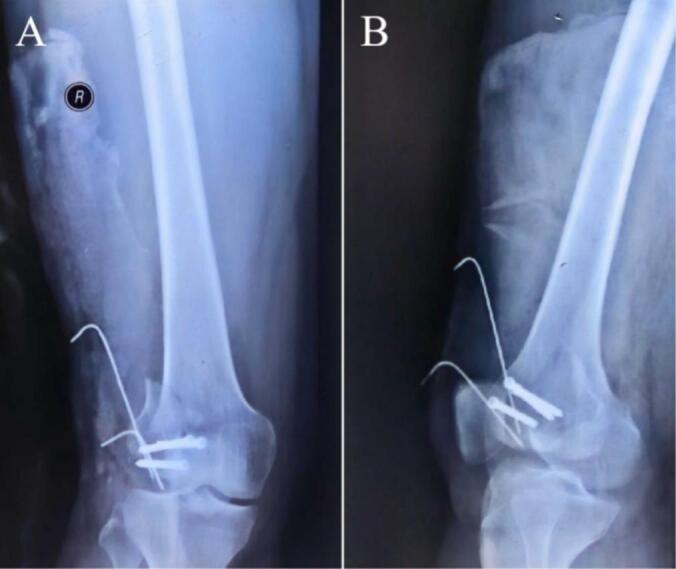
The X-ray images of initial incisional repositioning for internal fixation at a local hospital. (A) AP view of the right knee. (B) Lateral view of the right knee.

**Fig. 3:**
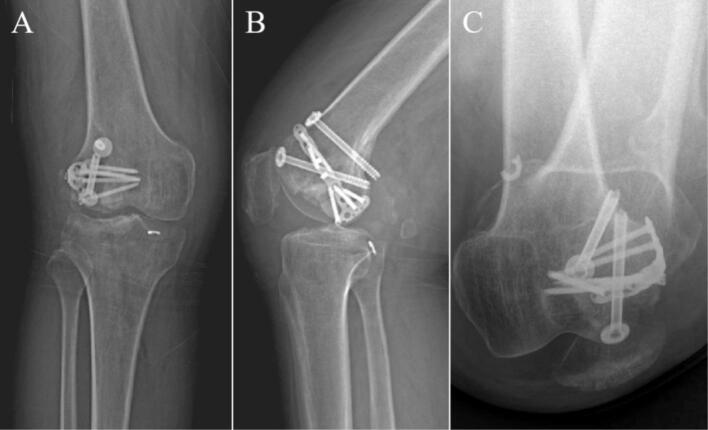
The X-ray images of a second incision for internal fixation at a local hospital. (A) AP view of the right knee. (B) Lateral view of the right knee. (C) Axial view of the right knee.

**Fig. 4:**
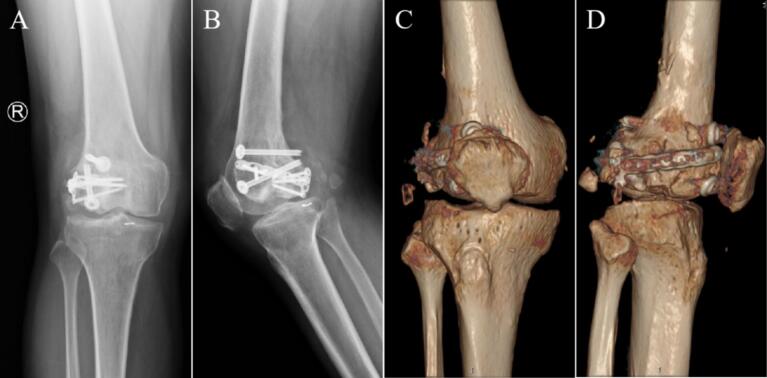
The preoperative images at our hospital. (A) AP view of the right knee X-ray. (B) X-ray lateral view of the right knee. (C) CT reconstruction AP view of the right knee. (D) three-dimensional CT reconstruction lateral view of the right knee.

A 52-year-old man suffered a fracture of the right distal femur due to a fall from height 22 months earlier (Fig. 1). At that time, internal fixation was performed at a local hospital. The surgical incisions healed well (Fig. 2). However, during a postoperative follow-up two months later, it was noted that the bone healing was inadequate, with fracture displacement resulting from unstable internal fixation. The patient then underwent revision surgery that included additional internal fixation and iliac bone grafting at another local facility, accompanied by regular postoperative follow-ups (Fig. 3).Currently, X-rays of the right distal femur taken at our hospital indicated postoperative changes attributed to the previous internal fixation, as well as the presence of hardware (Fig. 4). The diagnosis was established as postoperative malunion of the Hoffa fracture of the right distal femur with secondary traumatic arthritis.

Physical Examination Findings:

Slight swelling of the right knee joint.
Visible surgical scar.
Local tenderness upon palpation (VAS score: 7/10).
Restricted ROM: 0°–60° flexion.
Unstable knee valgus.

The patient had a medical history of hypertension, which had been well-controlled with regular oral administration of indapamide sustained-release tablets for the past four years. Preoperative examinations did not reveal any significant abnormalities. The patient expressed a strong desire to proceed with surgery, with no apparent contraindications identified. Both the patient and his family were informed of the nature of the condition and the associated surgical risks. The planned surgical procedures included the removal of the internal fixation hardware, debridement of cartilage, and right lateral UKA. Informed consent was obtained from the patient and his family prior to the procedure.

### 2.1 Preoperative preparation

**Fig. 5:**
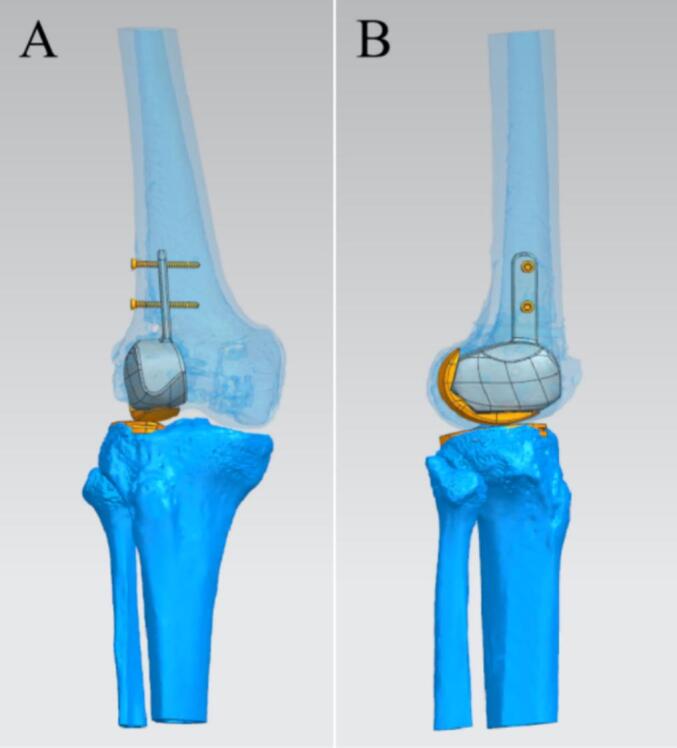
The images of the preoperative design of the customized prosthesis. (A) AP view of the right knee. (B) Lateral view of the right knee.

Low molecular weight heparin sodium was routinely used to prevent VTE after admission. Thorough preoperative assessments were conducted, including X-rays and CT reconstruction of the right knee joint. DVT showed no thrombosis in both lower limbs. Because the medial condyle was fine, TKA was not considered. Secondly, considering the age of the patient, the need to preserve the lateral cortical bone during the operation to maintain the attachment of the lateral collateral ligament and the postoperative mobility is large, we selected a customized prosthesis that can accurately reconstruct the anatomy. Utilizing the CT three-dimensional reconstruction data from this patient, Mimics software (Materialise, Belgium) was employed to design a customized trial mold, a lateral femoral condylar prosthesis, a distal femoral hemiarticular surface prosthesis, and a hemitibial plateau prosthesis, in addition to a fixation plate (Fig. 5).

### 2.2 Surgical procedure

**Fig. 6:**
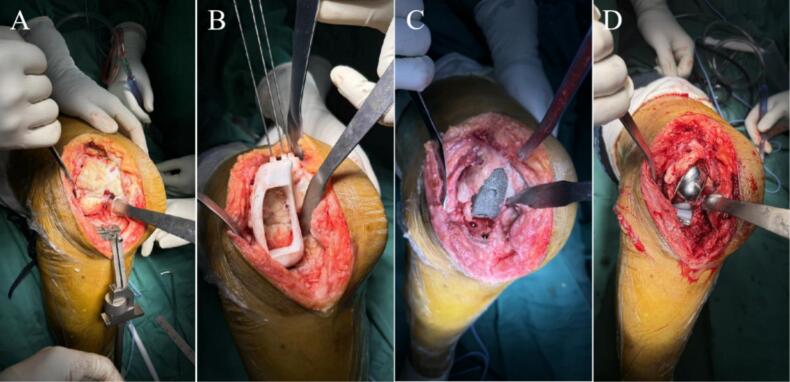
The intraoperative gross images. (A) Removal of the internal fixation. (B) Trial mold osteotomy. (C) Polishing the prosthetic space. (D) Fitting the customized prosthesis.

**Fig. 7:**
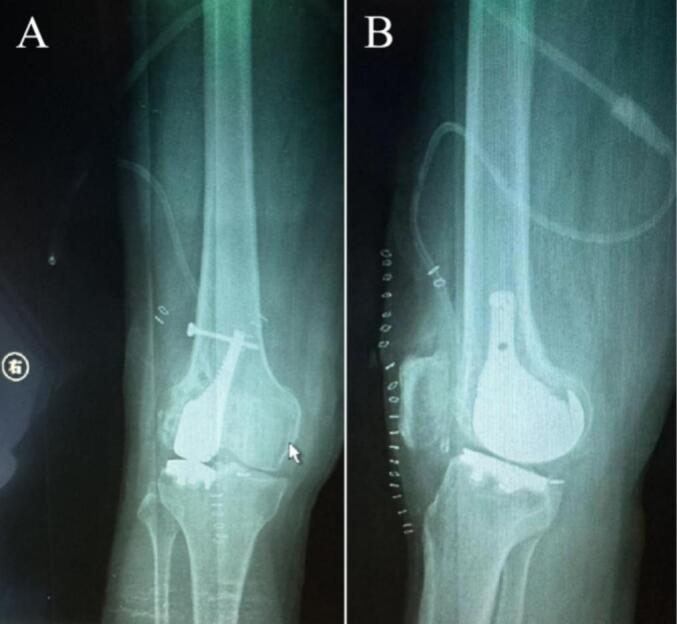
The intraoperative x-ray images. (A) AP view of the right knee. (B) Lateral view of the right knee.

Following successful administration of intraspinal anesthesia, the patient was placed in a supine position. Routine disinfection was performed, sterile drapes were applied, and the right lower limb was covered with a disposable, blood-repellent hemostatic collar. An anterolateral approach to the right knee was employed, with the incision made slightly laterally. Soft tissues were meticulously peeled layer by layer until the bone surface was exposed, revealing the metal internal fixation. The metal internal fixation was removed, revealing malunion of the lateral femoral condyle with destruction and collapse of the articular surface, accompanied by severe degeneration. Portions of the degenerated cartilage surface were excised. An osteotomy of the lateral femoral condyle and lateral tibial plateau was performed. A trial mold was fitted while preserving the lateral cortex, and the prosthetic space was prepared through chiseling and grinding. The preoperatively designed lateral unicondylar customized knee prosthesis, along with the distal femoral hemiarticular surface and hemitibial plateau prosthesis, were fitted, and the alignment of the force vector was adjusted (Fig. 6). Intraoperative X-rays were obtained to confirm the position of the prosthesis, determine the final placement configuration, and secure the prosthesis with a customized steel plate (Fig. 7). The prosthesis was secured using a cementless fixation method, allowing for optimal osseointegration. Following this, an assessment of knee joint mobility was conducted to evaluate ROM and stability. After thorough irrigation, drains were placed, and the surgical incision was repaired and sutured layer by layer before closing the wound. The number of gauze and instruments used during the procedure was counted, the blood-repellent hemostatic collar was loosened, and the surgery was completed.

### 2.3 Postoperative results

**Fig. 8:**
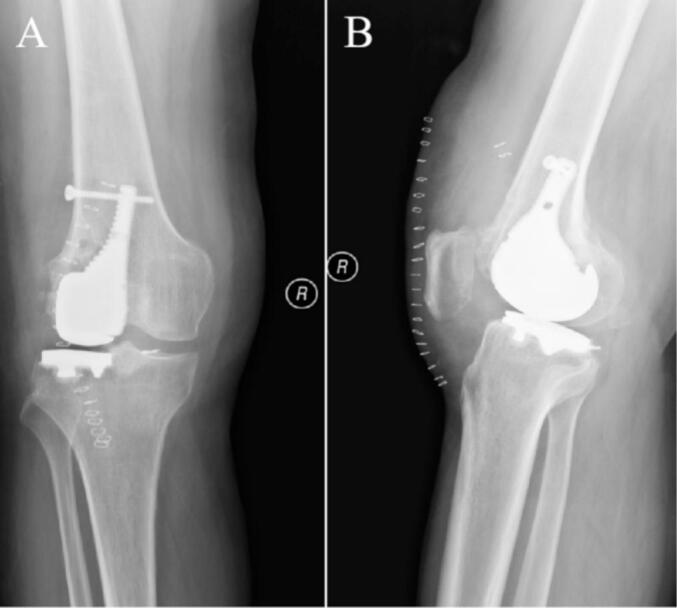
The pre-discharge X-ray images. (A) AP view of the right knee. (B) Lateral view of the right knee.

The patient successfully underwent right lateral UKA, which lasted for 3 h, with an estimated blood loss of approximately 200 ml. Intraoperative X-rays confirmed that the position of the prosthesis was satisfactory, and no significant adverse events were observed during the procedure. Immediately postoperatively, the patient was instructed to initiate functional rehabilitation exercises. The wound healed well, with no apparent complications. The drainage tube was removed on the 9th postoperative day, and the correct positioning of the prosthesis was verified through X-rays examination before the patient was discharged from the hospital (Fig. 8).

**Fig. 9:**
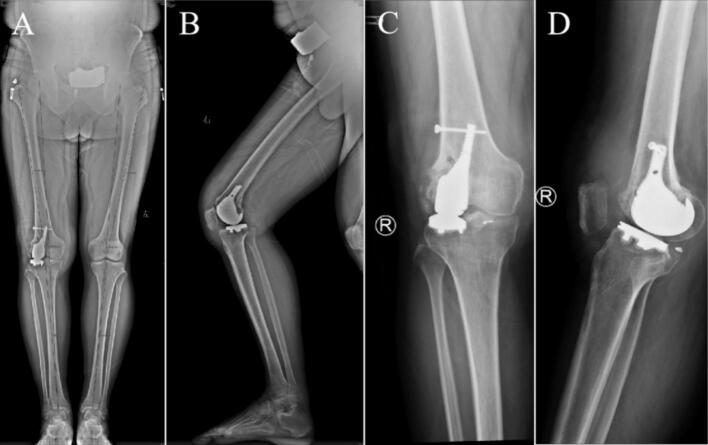
The X-ray images at eighth postoperative week. (A) Full-length AP view of both lower extremities. (B) Lateral view of the right lower extremity. (C) AP view of the right knee. (D) Lateral view of the right knee.

**Fig. 10:**
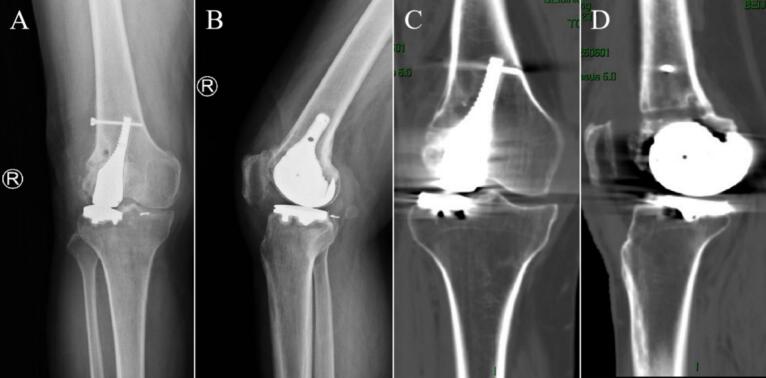
The images at twelfth postoperative weeks. (A) AP view of the right knee X-ray. (B) Lateral X-ray of the right knee. (C) CT AP view of the right knee. (D) CT lateral view of the right knee.

**Fig. 11:**
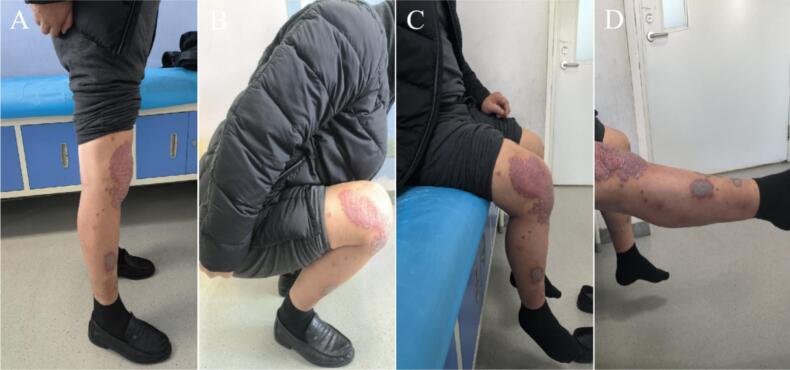
At twelfth postoperative weeks, the patient's knee ROM. (A) Standing lateral view. (B) Squatting lateral view. (C) Sitting lateral view. (D) Sitting position with the affected limb in the extended position.

Eighth postoperative week, the right knee joint demonstrated good mobility. Both internal and external rotation stress tests yielded negative results, indicating joint stability. X-rays at this follow-up revealed an appropriate force line of the lower limb and proper positioning of the joint prosthesis (Fig. 9). Twelfth weeks after the operation, a review was performed, which included X-rays, CT and the patient's ROM; the X-rays and CT showed that the position of the prosthesis and the line of force of the lower extremity were still good (Fig. 10, Fig. 11). The patient achieved pain resolution (VAS: 1/10) and significant functional improvement (KSS: 85/100; ROM: 0°–125° flexion), no complications occurred.

## 3 Discussion

Hoffa fractures are clinically characterized as high-energy injuries that primarily affect young adults [14]. The injury typically results from axial shear forces acting on the posterior femoral condyle while the knee is flexed [15]. Specifically, the axial force from the proximal femur is transmitted through the femoral condyles to the distal tibia and tibial plateau, particularly during emergency braking when the knee is in a flexed or valgus position. This scenario can produce significant shear forces between the femoral condyles and the tibial plateau, precipitating Hoffa fractures [16]. Such injuries are often associated with high-impact events, such as motor vehicle accidents (especially motorbike collisions) and falls from significant heights [17].

In the case of isolated Hoffa fractures of the lateral condyle, the anterolateral patellar approach is commonly employed for incisional reduction and internal fixation. This approach provides adequate exposure of the fracture site while minimizing the risk of damage to nerves and blood vessels [18]. While traditional internal fixation methods can yield favorable results, their limitations are becoming more evident in elderly patients, even with simple Hoffa fractures. Currently, joint replacement is recognized as an effective treatment for malunion following distal femur fractures. Both TKA and UKA are viable options [19]. Research indicates that medial UKA often provides superior outcomes and is more widely employed in clinical settings globally; in contrast, lateral UKA has been reported less frequently, accounting for approximately 1 % of all knee arthroplasties [20,21]. The use of customized UKA prostheses is based on the comprehensive consideration of the age of the patient, the retention of the lateral skin during the operation to maintain the attachment of the lateral collateral ligament, and the choice of the patient to have a large amount of activity after the operation. There are fewer studies related to the use of customized prostheses, and we see some short-term advantages, but this also requires us to monitor the results of knee follow-up over time and compare them with incisional resurfacing, internal fixation, or TKA to assess the exact prognosis.

In the present case, the patient underwent lateral UKA due to postoperative malunion of a Hoffa fracture of the distal femur, with an accompanying diagnosis of traumatic arthritis. The surgical technique employed in this case preserved the lateral cortex, thereby maintaining the attachment site for the lateral collateral ligament. Additionally, a cementless anchored customized prosthesis was utilized in the procedure. This approach offered significant anatomical advantages, providing immediate and reliable stability that facilitated early postoperative activities, including routine motions and partial weight-bearing exercises. The capacity to resume these activities shortly after surgery has not been extensively reported in the literature, thus highlighting the innovative and informative nature of this case. The short-term efficacy of customized prosthesis is excellent, but the long-term complications such as prosthesis loosening, wear and periprosthetic fracture need to be paid attention to during long-term follow-up [22].

The primary advantage of customized prostheses is their capacity for precise design and manufacturing tailored to the patient's unique anatomy and needs [23]. Compared to standard prostheses, customized options can ultimately provide a better fit, reduce the risk of postoperative complications, and offer enhanced biomechanical support [24]. In this case, follow-up imaging and functional evaluations indicated that the patient experienced significant pain reduction and satisfactory recovery of knee mobility, underscoring the effective adaptability of the customized prosthesis.

Despite the positive outcomes reported in this case, several challenges warrant consideration for the broader clinical application of customized prostheses. First, the cost of these prostheses, which typically falls outside of health insurance coverage, may limit their accessibility for many patients due to the out-of-pocket expenses associated with their use. Second, the long-term effects and overall effectiveness of customized prostheses in the context of UKA still necessitate validation through extensive, multicenter clinical trials. More importantly, Future studies should focus on evaluating the applicability and long-term outcomes of customized prostheses across diverse patient demographics to facilitate better-informed clinical practices.

## 4 Conclusion

The implementation of customized prostheses in lateral UKA presents a promising approach to addressing postoperative malunion of distal femur fractures. This case highlights a rare occurrence of postoperative malunion and traumatic arthritis following a Hoffa fracture of the distal femur, treated effectively through lateral UKA using a customized prosthesis.

## Patient consent

Written informed consent was obtained from the patient for the publication of this case report and accompanying images.

## Ethical approval

This study was approved by the Institutional Review Board of Beijing Jishuitan Hospital, Capital Medical University(No. 2020–15).

## Guarantor

Wei Han

## Funding

This study is financially supported by the Beijing Natural Science Foundation-Haidian Original Innovation Joint Foundation (No. L242068) and the 10.13039/501100012166National Key Research and Development Program (No. 2024YFB3613303).

## Registration of research studies

Not applicable.

## Declaration of competing interest

No potential conflicts of interest were disclosed.

## References

[1] Heuschen U.A., Gohring U., Meeder P.J. (1994). Bilateral Hoffa fracture—a rarity. Aktuelle Traumatol..

[2] Bartonicek J., Rammelt S. (2015). History of femoral head fracture and coronal fracture of the femoral condyles. Int. Orthop..

[3] Letenneur J. (1978). Hoffa’s fractures. Report of 20 cases (author’s transl). Ann. Chir..

[4] Kumar R., Malhotra R. (2001). The Hoffa fracture: three case reports. J. Orthop. Surg. (Hong Kong).

[5] Orapiriyakul W., Apivatthakakul T., Buranaphatthana T. (2018). How to determine the surgical approach in Hoffa fractures?. Injury.

[6] Zhou Y. (2019). Hoffa fracture of the femoral condyle: injury mechanism, classification, diagnosis, and treatment. Medicine (Baltimore).

[7] Dhillon M.S. (2012). Coronal fractures of the medial femoral condyle: a series of 6 cases and review of literature. Musculoskelet. Surg..

[8] Meyer C. (2004). Difficulties involved in the Hoffa fractures. Unfallchirurg.

[9] Tetsunaga T. (2013). Posterior buttress plate with locking compression plate for Hoffa fracture. J. Orthop. Sci..

[10] Tack P. (2016). 3D-printing techniques in a medical setting: a systematic literature review. Biomed. Eng. Online.

[11] Levengood G.A., Dupee J. (2018). Accuracy of coronal plane mechanical alignment in a customized, individually made Total knee replacement with patient-specific instrumentation. J. Knee Surg..

[12] Belzile E.L., Angers M., Bedard M. (2020).

[13] Sohrabi C. (2023). The SCARE 2023 guideline: updating consensus Surgical CAse REport (SCARE) guidelines. Int. J. Surg..

[14] Jain A., Aggarwal P., Pankaj A. (2014). Concomitant ipsilateral proximal tibia and femoral Hoffa fractures. Acta Orthop. Traumatol. Turc..

[15] Gavaskar A.S., Tummala N.C., Krishnamurthy M. (2011). Operative management of Hoffa fractures--a prospective review of 18 patients. Injury.

[16] Kondreddi V., Yalamanchili R.K., Ravi Kiran K. (2014). Bicondylar Hoffa's fracture with patellar dislocation - a rare case. J. Clin. Orthop. Trauma.

[17] Arastu M.H. (2013). Coronal plane partial articular fractures of the distal femoral condyle: current concepts in management. Bone Joint J..

[18] Tong W. (2014). Efficacy of multiple Acutrak hollow headless compression screws in the treatment of Hoffa fractures. Chin. J. Orthop..

[19] Price A.J. (2018). Knee replacement. Lancet.

[20] Stuyts B. (2015). Custom-made lateral femoral hemiarthroplasty for traumatic bone loss: a case report. Knee.

[21] Smith J.R. (2014). Fixed bearing lateral unicompartmental knee arthroplasty--short to midterm survivorship and knee scores for 101 prostheses. Knee.

[22] Chen L. (2015). Indications, outcomes, and complications of unicompartmental knee arthroplasty. Front. Biosci. (Landmark Ed.).

[23] Brinkmann E.J., Fitz W. (2021). Custom total knee: understanding the indication and process. Arch. Orthop. Trauma Surg..

[24] Moret C.S. (2021). Customised, individually made total knee arthroplasty shows promising 1-year clinical and patient reported outcomes. Arch. Orthop. Trauma Surg..

